# Modulation of piglets’ microbiota: differential effects by a high wheat bran maternal diet during gestation and lactation

**DOI:** 10.1038/s41598-017-07228-2

**Published:** 2017-08-07

**Authors:** Julie Leblois, Sébastien Massart, Bing Li, José Wavreille, Jérôme Bindelle, Nadia Everaert

**Affiliations:** 1 Precision Livestock and Nutrition Unit, Gembloux Agro-Bio Tech, TERRA, Teaching and Research Centre, University of Liège, 5030 Gembloux, Belgium; 2 Research Foundation for Industry and Agriculture, National Scientific Research Foundation (FRIA-FNRS), Brussels, Belgium; 3 Laboratory of Urban and Integrated Plant Pathology, Gembloux Agro-Bio Tech, TERRA, Teaching and Research Centre, University of Liège, 5030 Gembloux, Belgium; 4 Production and Sectors Department, Walloon Agricultural Research Centre, 5030 Gembloux, Belgium

## Abstract

Reaching a beneficial intestinal microbiota early in life is desirable for piglets, as microbiota will impact their future health. One strategy to achieve this is the addition of prebiotics to sows’ diet, as their microbiota will be transferred. Transmission of microbiota to the offspring occurs at birth and during lactation but a transfer might also occur during gestation. The objectives of this study were to determine whether and when (before and/or after birth) a maternal transfer of the microbiota occurs, and to observe the impact of wheat bran (WB) in sows’ diet on their faecal microbiota, their offspring’s microbiota and fermentation profile. Sequencing was performed on DNA extracted from umbilical cord blood, meconium, sows’ faeces and piglets’ colon content. Short-chain fatty acid production was determined in piglets’ distal gut. Different bacteria (mostly Proteobacteria, followed by Firmicutes) were found in the umbilical cord blood, suggesting a maternal transfer occurring already during gestation. Less butyrate was produced in the caecum of WB piglets and a lower concentration of valerate was observed in all intestinal parts of WB piglets. Maternal wheat bran supplementation affected microbiota of sows and piglets differently.

## Introduction

Intestinal microbiota is acquired early in life and plays multiple roles on host’s health: fermentation of fibrous dietary compounds, synthesis of vitamins, maturation of the gut associated lymphoid and immune tissues and resistance to pathogen colonization^1–5^. The fermentation of undigested carbohydrates by fibrolytic bacteria within the large intestine leads to the production of short-chain fatty acids (SCFA), mainly acetate, propionate and butyrate^6, 7^ that are used as energy sources by the host. In particular, butyrate is the main energy source for colonocytes and is considered health-promoting due to its anti-inflammatory properties^8^. An increase in gut butyrate production might improve host’s health and can be beneficial in pig production as piglets are prone to infections especially around the weaning period. In an attempt to apply such a strategy, Ivarsson *et al*.^9^ observed an increased ileal and faecal butyrate production when feeding growing pigs a high wheat bran (WB) diet (14%) in comparison with other fibre sources (pectin or arabinoxylan sources).

A second factor that might improve piglets’ health, possibly on the long term, is the establishment of a beneficial microbiota early in life. This might be done by modulating the sow’s microbiota that will be transferred to the offspring. This vertical transfer of the microbiota has been shown both in humans^10^ and in pigs^11, 12^. It takes place mainly at birth via a transfer of vaginal and faecal microbes from the mother. However, an earlier mechanism has been more recently unravelled. In humans, a transfer of bacteria has been proven to occur during gestation as bacteria were found in the umbilical cord blood, the meconium, the amniotic fluid^13, 14^ even if the digestive tract of new-borns had always previously been considered as sterile and firstly colonized at birth^7, 8^.

In order to take advantage of this interplay between sows and offspring for pig production purposes, some studies showed that prebiotics in the sow’s diet can improve piglets’ health status (i.e. greater levels of IgG, IFNγ and activated T cells) by using prebiotics^15, 16^ but few focussed on the direct impact of sows’ diet on the offspring’s microbiota^11, 12^.

Wheat bran (WB) is a source of insoluble non-starch polysaccharides, rich in arabinoxylans, cellulose and lignin that is commonly used in sows’ diets for its bulking properties and may be considered as a prebiotic due to its ability to be fermented in the large intestine^17–19^. As it has been shown that WB can induce microbiota and SCFA changes in growing pigs’ ileum and faeces^9^, WB in the maternal diet was used in this study to investigate whether an altered microbiota was observed in sows’ faeces at different time points and if this treatment could in turn affect the microbiota and SCFA production of their offspring. Moreover, to investigate *in utero* microbiota transfer, the umbilical cord blood and meconium were collected at birth and analysed for subsequent microbiota determination.

## Methods

### Animals

The animal experiment and all interventions on animals were approved by the ethical committee of the University of Liège (Belgium, licence number 1661 approved 31^st^ January 2015) and were in compliance with European (directive 2010/63/EU) and Belgian (C − 2013/24221, AR of 23^rd^ of March 2013) regulations concerning the use and care of animals for scientific purposes. The experiment was run at the Walloon Agricultural Research Centre in Gembloux (Belgium). Fifteen Landrace sows, inseminated with Piétrain semen, parity 1 to 5, were divided in two groups, equilibrated for parity, body weight and genetic background.

### Housing

Sows were housed in groups during the gestation period from 3 days after artificial insemination (AI) until 7 days before farrowing. Gestation pens used straw as bedding; the individual farrowing units used wood shavings as bedding.

### Diets and feeding

From day 3 after AI to day 43, all sows received the same gestation diet containing 7% of WB. At day 43, the sows were split in 2 groups and each group was assigned to a dietary treatment, either a control diet (CON, N = 7) or a wheat bran-based diet (WB, N = 8) until the end of the lactation period (28 days after farrowing). Day 43 was chosen to allow a long adaptation period to the sows and because ultrasound had been performed to confirm gestation of all sows. The same ingredients were used for both the CON and WB diet. WB diet contained 250 g/kg DM of wheat bran during gestation and 140 g/kg DM during lactation. For a similar feeding phase, diets of both groups were formulated to supply similar amounts of net energy and protein. The composition and nutritive values are given in Supplementary Table ST1. Sows were restrictively fed during the gestation period and fed *ad libitum* during the whole lactation period, diets being adapted to their nutritional requirements at each feeding phase (gestation and lactation). Piglets had access to creep feed during the lactation period. The creep feed was devoid of wheat bran, non-starch polysaccharidases and organic acids (composition displayed in Supplementary Table ST2).

### Sample collection

Faeces were collected directly from the rectum of the sows during gestation, 21 days after AI (G21) and 98 days after AI corresponding to 16 days before farrowing (G98+), respectively before and after the dietary change that took place on day 43. Sows’ faeces were also collected during the lactation period, i.e. 20 days post-farrowing (L). Faecal samples were placed immediately in sterile bags, snap-frozen and stored at −80 °C until DNA extraction. Farrowing was induced by the injection of 2 ml of sodium cloprostenol (92 µg/ml) at 114 days of gestation. For one piglet during each farrowing, a 5 ml sample of umbilical cord blood was collected with a sterile syringe and tube by clamping the cord while the piglet was being born. The same piglet was directly removed from the sows’ vulva and euthanized in order to collect meconium in the colon that was snap-frozen and stored at −80 °C until DNA extraction. Fourteen blood and meconium samples were collected in total (6 from CON sows, 8 from WB sows). On days 26 and 27 of lactation, 8 female piglets per group (16 in total, 2 piglets/sow, 4 sows/treatment) were euthanized. A mix of Xylazine/Zoletil 100 (4 mg of xylazine, 2 mg of zolazepam and 2 mg of tilamine/kg) was used for anaesthesia followed by T-61 injection directly in the heart (0.1 ml/kg) for euthanasia. Their ileal, caecal and colonic contents were immediately collected in sterile tubes, snap-frozen and stored at −80 °C until further analysis.

### DNA extraction and sequencing

DNA from sows’ faeces, piglets’ meconium and colon content was extracted with the QIAamp DNA Stool Mini Kit (Qiagen, Hilden, Germany), following the manufacturer’s instructions modified by the addition of two bead-beating steps (FastPrep-24, MP Biomedicals, Illkirsh, France), as described by Yu and Morrisson^20^. DNA from umbilical cord blood was extracted with QIAamp DNA Blood Mini Kit (Qiagen, Hilden, Germany). The concentration and quality of the DNA were confirmed on a Nanodrop (Thermo Scientific NanoDrop 2000, USA) and by an agarose gel (1%). DNA was then stored at −20 °C until sequencing. Sequencing was performed by DNAVision (Gosselies, Belgium), using the Illumina MiSeq (2 × 300nt) and after amplifying, purifying and tagging the hypervariable regions V3-V4 (Forward primer: 5′-TCGTCGGCAGCGTCAGATGTGTATAAGAGACAGCCTACGGGNGGCWGCAG-3′ and reverse primer: 5′- GTCTCGTGGGCTCGGAGATGTGTATAAGAGACAGGACTACHVGGGTATCTAATCC-3′) following the 16 S Metagenomic Sequencing Library Preparation protocol (Part # 15044223 Rev. B) from Illumina. For sows’ faecal DNA, 6 DNA samples per treatment were analysed by sequencing to exclude samples of sows with high parity. For piglets, 7 samples per maternal treatment were analysed based upon the need of high quality DNA for sequencing.

### Short-chain fatty acids (SCFA) and branched-chain fatty acids (BCFA) determination

Piglet’s intestinal content and sow’s faeces were diluted in ultrapure water to obtain a 6-fold dilution prior to determination of SCFA and lactate by high performance liquid chromatography (HPLC). Piglets’ short chain and branched chain fatty acids were analysed by isocratic HPLC, using a Waters system fitted with an Aminex HPX-87H column (Bio-Rad, Hercules, CA, USA) combined with a UV detector (210 nm) with sulfuric acid (5 mM) as mobile phase at a flow rate of 0.6 ml/min. Each peak was integrated by the Empower 3 software (Waters, Milford, USA) after the encoding of the standard curve and then verified manually. The results were expressed in mg.ml^−1^ and were transformed in mg.g^−1^ and mmol.g^−1^, taking into account the initial dilution. The percentages of SCFAs (acetate, propionate, butyrate and valerate), BCFAs (isobutyrate, isovalerate), and lactate were calculated based on the molar ratios. The variable number of samples observed for different intestinal parts is explained by the lack of intestinal contents of some pigs at slaughtering.

### Bioinformatics and statistical analyses

Raw sequences of 16S rRNA were assigned to each sample, quality checked and trimmed using Basespace default parameters (Illumina). Sequences were assigned to 97% ID OTUs by comparison to the Greengenes reference database 13.8 using the QIIME (Quantitative Insights Into Microbial Ecology) 1.9.0 software. Since samples contained variable number of sequences (mean ± SEM of 35893 ± 5552 for sows’ faeces, 23440 ± 3747 for piglets’ colon contents and 209 ± 90 for the umbilical cord blood), diversity analyses were carried out on samples rarefied at the same sequencing depth to avoid bias in sequencing depth between samples. The low number of sequences for the umbilical blood was probably due to the low numerical count of bacteria present in blood in opposition with intestinal contents, as already observed by Vientós-Plotts *et al*.^21^. The Beta_diversity_through_plots.py script was used to assess differences in bacterial communities and functional composition between groups of samples. Beta diversity was visualized using un-weighed, weighed UniFrac and Bray–Curtis distances with Principal Coordinate Analysis (PCoA). The compare_categories.py script, which applied the adonis method on the previously obtained dissimilarity matrices, was used to determine whether communities differed significantly between groups of samples. Multiple_rarefactions.py and alpha_diversity.py scripts were applied to compute alpha diversity metrics, which evaluated diversity within a sample and generated rarefaction curves. All statistical analyses were performed with SAS 9.2 software (Cary, NC USA). Microbiota results were analysed with the Kruskall-Wallis test which is a non-parametric analysis of variance including multiple comparisons. P-values and false discovery rate (FDR) corrections were determined by the MULTTEST procedure of SAS that calculates the adjusted p-value by using the linear method of Benjamini and Hochberg. SCFA, BCFA and lactate results were analysed with the proc MIXED of SAS, using the treatment alone (piglets) or treatment and period (sows) as fixed factors. Normality of data (Shapiro-Wilk’s test) and variance equality (Levene’s test) were checked in SAS prior to analysis. Pearson’s correlation coefficient between SCFA ratios and microbiota in colon content were calculated using the PROC CORR of SAS. All data were presented as mean and the SCFA data was presented as mean ± SEM; for all analyses, differences were considered as significant when p-value < 0.05 and as substantial when p-values < 0.1.

## Results

**Figure 1:**
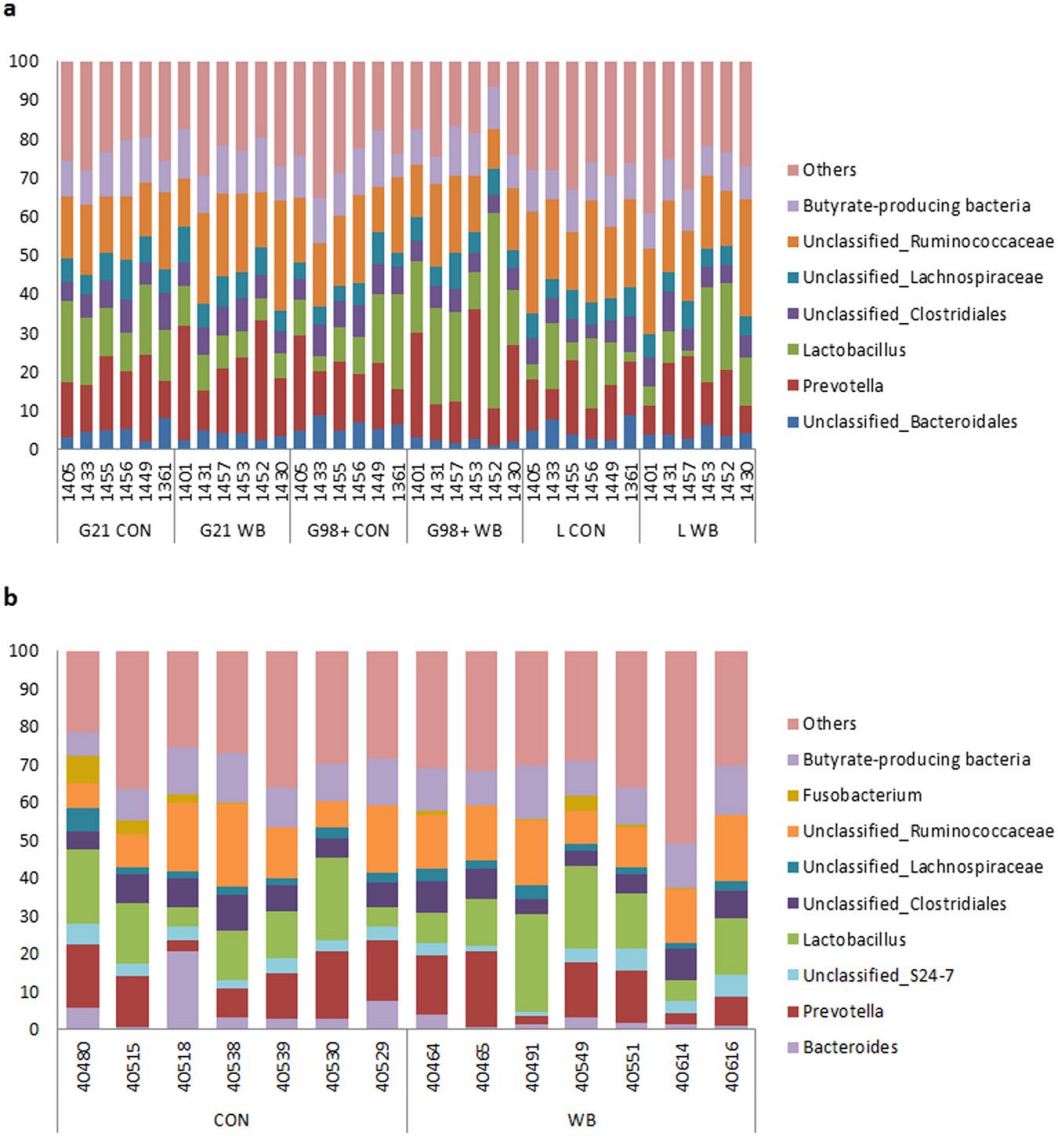
Individual composition of sows’ (**a**) and piglets’ (**b**) microbiota at the genus level. The Y-axis represents the relative abundances of the different genera (expressed as % of the total microbiota) and the X-axis represents the individuals (ID number).

Results of the individual composition of sows’ (a) and piglets’ (b) microbiota are presented in Fig. 1, with a grouping of butyrate-producing bacteria. On the X-axis, each bar chart represents individual animals (ID number on the axis). Because of the important role of butyrate in health-promoting mechanisms of intestinal microbiota, specific attention was paid to the butyrate-producing bacteria in the microbiota analyses. These butyrate-producing bacteria group includes the *Clostridium, Anaerostipes, Blautia, Butyrivibrio, Coprococcus, Dorea, Lachnospira, Pseudobutyrivibrio, Roseburia, Faecalibacterium, Oscillospira, Ruminococcus, Megasphaera* and *Butyricimonas* genera. This group is not exhaustive is based on the classification provided by several articles found in litterature^22–24^. What is clear from this figure is that there exists a large variability between individual sows even within the same group. To illustrate this, *Prevotella* (in red) can vary for one group (G98+CON e.g.) from 9% to 25% of the total microbiota. For piglets, the same tendency is observed (from 3% to 16% of the total microbiota is *Prevotella* in the CON group). The same observations can be made for several groups (*Lactobacillus, Bacteroides*).

### Sows

**Figure 2:**
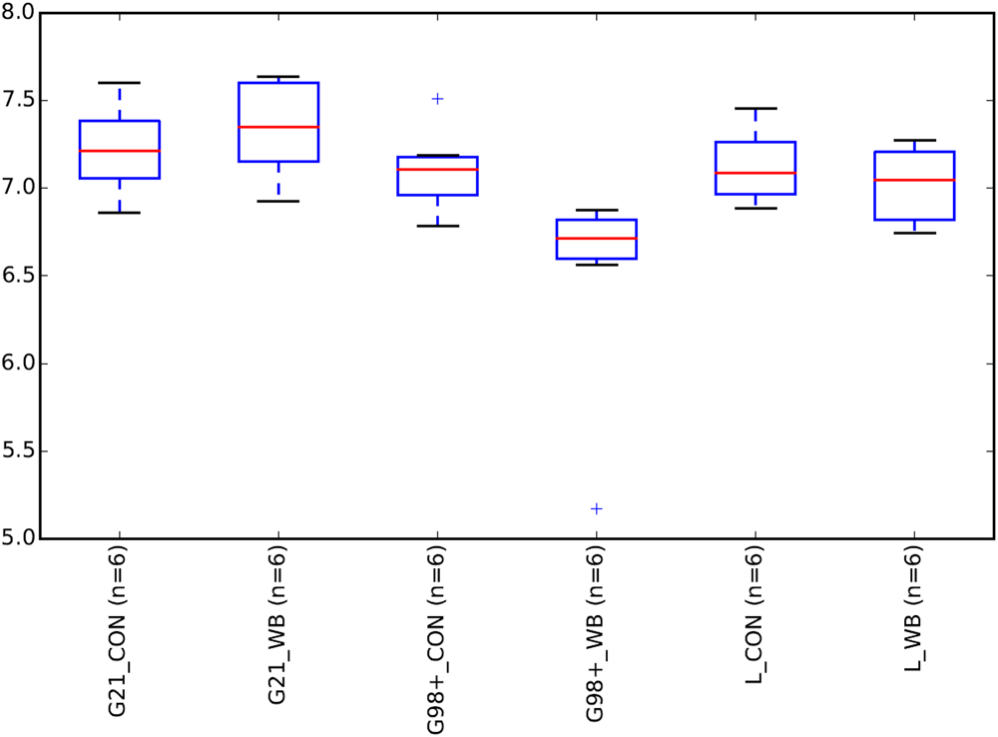
Distribution of alpha diversity as measured by Shannon index, box plots represent the calculated Shannon index for microbiota samples of sows fed the control diet (CON, N = 6) and the wheat bran-enriched diet (WB, N = 6) at three different stages: 21 d (G21) and 98 d (G98+) of gestation, respectively before and after the experimental diets were distributed, and 20 d of lactation (L).

**Figure 3:**
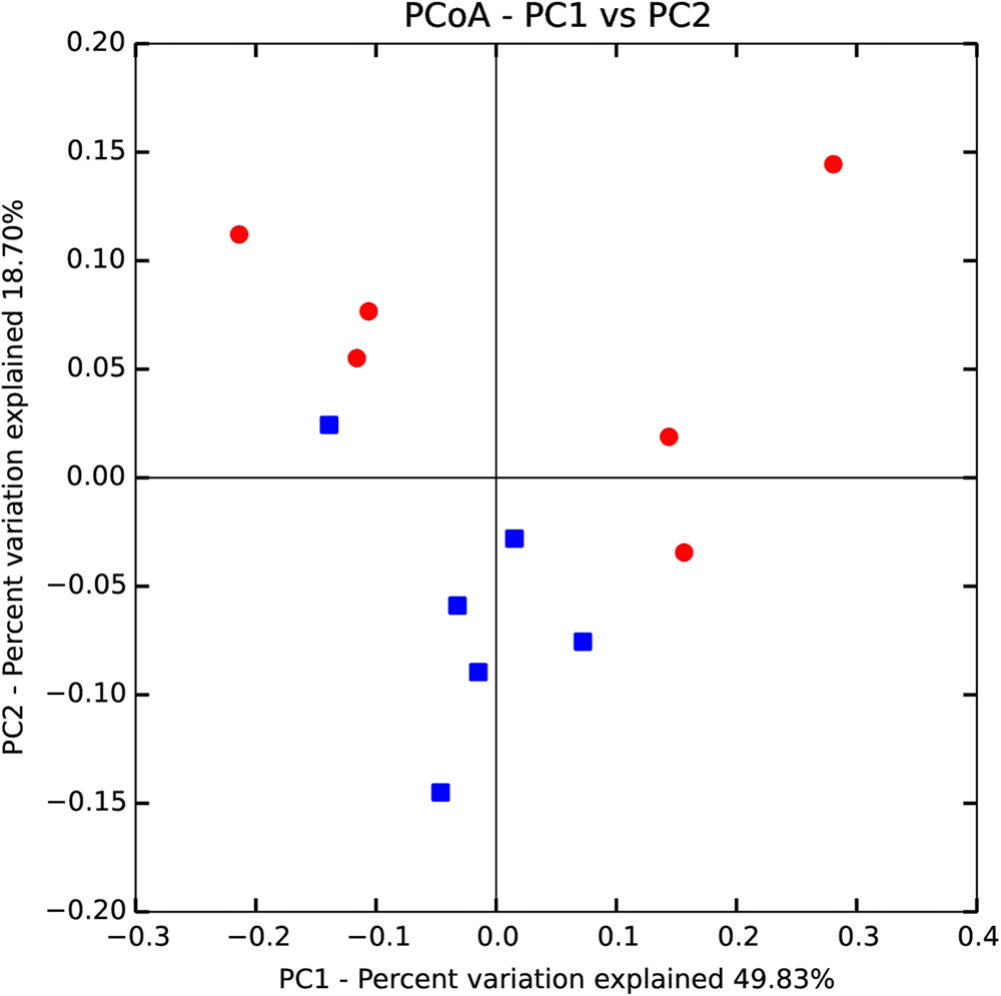
PCoA of microbial communities of sows fed the control diet (CON, N = 6) and the wheat bran-enriched diet (WB, N = 6) 98d (G98+) into gestation. Individual WB sows are displayed in red and CON sows in blue.

The box plot (Fig. 2) shows a numerical difference in the calculated Shannon index between CON (7.10) and WB (6.48) samples during G98+, although the Shannon index was not significantly different (p-value = 0.06). During G21 and L, the p-value between groups were not significant (p-value = 1). The PCoA for sows sampled during gestation when fed the experimental diets shows a clear separation between the CON and the WB groups, the two axes explaining 68% of the total variability (Fig. 3, PCoA based on the weighted Unifrac distance). Such clustering could not be found during the lactation period (see Supplementary Figure SF1).

During the gestation period (G98+), 13 genera differed in relative abundance (P < 0.05) between the CON and the WB group (Table 1), probably driving the PCoA separation. Most of the genera for which the raw p-value was significant showed a higher abundance in the CON than in the WB group (including *Parabacteroides*, Unclassified_*Bacteroidales*, Unclassified_RF16, Unclassified_*Clostridiales* and *Oscillospira*, a butyrate-producing bacterium), while only Unclassified_*Erysipelotrichaceae* OTU1 were more abundant in the WB group. Only 3 genera showed a significant difference with FDR correction (FDR < 0.05, *Parabacteroides*, Unclassified_*Bacteroidales*, SMB53). During lactation (L), two genera, the unclassified RF32 and *Ruminobacter*, showed significant differences (p < 0.05) between groups, these two being higher in the WB group. It is worth noting that some bacteria, even though non-significantly different between both groups show a numerical difference with a p-value < 0.10. Indeed, the butyrate-producing bacterium *Butyrivibrio* is higher in the WB group in lactation compared to the CON group. Before the separation of the two dietary groups (G21), the microbiota of the sows was also analysed. The results can be found in the Supplementary Table ST3. Minor genera (<1% of the total microbiota) were different before the dietary change but were not different afterwards. For *Lactobacillus*, a significant difference was observed: the future CON group had a higher abundance than the future WB group, which is the opposite of what we can observe numerically during G98+.

**Table 1:** Composition of the faecal microbiota of sows fed the control diet (CON, N = 6) and the wheat bran-enriched diet (WB, N = 6) at two different stages: 98 d into gestation (G98+) and 20 d into lactation (L), expressed as a percentage (%) of the total microbiota.

	G98+				L			
	CON (N = 6)	WB (N = 6)	P-values	FDR	CON (N = 6)	WB (N = 6)	P-values	FDR
Bacteroidetes	29.3	25.4	NS	NS	23.2	22.7	NS	NS
*Parabacteroides*	0.36	0.14	<**0**.**001**	**0**.**02**	0.36	0.32	NS	NS
Unclassified_*Bacteroidales*	6.13	2.25	<**0**.**001**	**0**.**02**	5.10	4.11	NS	NS
*Bacteroides*	0.22	0.04	<**0**.**005**	NS	0.12	0.11	NS	NS
CF231	1.22	0.57	**0**.**01**	NS	0.74	0.69	NS	NS
Unclassified_RF16	2.38	0.79	**0**.**03**	NS	0.78	0.43	NS	NS
*Prevotella*	15.5	19.0	NS	NS	12.7	13.7	NS	NS
Cyanobacteria	0.00	0.00	NS	NS	0.07	0.14	0.06	NS
Unclassified_YS2	0.11	0.14	NS	NS	0.07	0.14	0.06	NS
Firmicutes	63.9	67.8	NS	NS	71.2	68.5	NS	NS
*Butyrivibrio*	0.00	0.04	NS	NS	0.04	0.14	0.09	NS
SMB53	0.29	0.13	<**0**.**001**	**0**.**03**	0.83	0.80	NS	NS
Unclassified_*Lachnospiraceae* OTU2	0.09	0.02	<**0**.**005**	NS	0.04	0.03	NS	NS
Unclassified_*Clostridiales*	7.17	5.41	<**0**.**005**	NS	6.23	6.46	NS	NS
Unclassified_ *Erysipelotrichaceae* OTU1	0.02	0.06	**0**.**01**	NS	0.07	0.06	NS	NS
*Anaerovibrio*	0.20	0.53	**0**.**03**	NS	0.30	0.44	NS	NS
*Turicibacter*	0.13	0.07	**0**.**03**	NS	0.17	0.12	NS	NS
*Oscillospira*	2.69	1.76	**0**.**03**	NS	1.88	1.85	NS	NS
Unclassified_*Erysipelotrichaceae* OTU2	0.08	0.03	0.06	NS	0.05	0.05	NS	NS
Unclassified_*Mogibacteriaceae*	0.75	0.44	0.07	NS	0.46	0.61	NS	NS
*Lactobacillus*	12.2	23.4	NS	NS	9.47	12.3	NS	NS
*Streptococcus*	4.84	1.45	NS	NS	6.17	3.72	NS	NS
Unclassified_*Lachnospiraceae* OTU1	5.07	6.19	NS	NS	6.26	5.50	NS	NS
Unclassified_*Ruminococcaceae*	17.5	15.8	NS	NS	21.4	20.3	NS	NS
Unclassified_*Christensenellaceae*	0.55	0.52	NS	NS	3.20	3.02	NS	NS
Proteobacteria	1.28	1.50	NS	NS	1.57	2.49	NS	NS
Unclassified_*Enterobacteriaceae*	0.04	0.01	**0**.**01**	NS	0.31	0.43	NS	NS
*Ruminobacter*	0.02	0.03	NS	NS	0.00	0.01	**0**.**04**	NS
Unclassified_RF32	0.04	0.03	NS	NS	0.02	0.04	**0**.**05**	NS
Spirochaetes	3.16	3.29	NS	NS	2.30	3.80	NS	NS
Unclassified_Sphaerochaeta	0.33	0.12	0.07	NS	0.18	0.17	NS	NS
*Treponema*	2.83	3.17	NS	NS	2.12	3.63	0.09	NS

Only genera with a relative abundance >0.01% were included in this table. P-values and FDR were considered as non-significant (NS) when p > 0.1 and consistent when 0.05 < p < 0.1.

The SCFA molar ratios of the sows’ faeces (see Supplementary Table ST4) were not affected by the dietary treatment whatever the period, while a period-effect was observed (p < 0.001) for the total SCFA concentration. This value was higher during lactation than gestation.

### Umbilical cord blood and meconium

After DNA extraction on the meconium of the new-born piglets, bacterial DNA concentrations were below detection limits and thus could not be sequenced. Consequently, no meconium results can then be presented here. For umbilical cord blood, concentrations of >20ng/µl of DNA were reached for every sample and underwent sequencing. Shannon indexes did not differ between treatments (boxplot displayed in Supplementary Figure SF1, p = 0.4). Results (Table 2) showed that different bacteria are present in the umbilical cord blood. Proteobacteria accounted for 51.6% and 46.6% of the total bacteria present in the CON and the WB groups, respectively. The second most abundant phylum was Firmicutes while Actinobacteria and Bacteroidetes were also well represented.

**Table 2:** Microbial composition of the umbilical cord blood of piglets born from sows fed the control diet (CON, N = 6) and the wheat bran-enriched diet (WB, N = 8), expressed as the percentage (%) of the total microbiota.

	CON (N = 6)	WB (N = 8)	p-value	FDR
Actinobacteria	12.4	8.7	NS	NS
*Corynebacterium*	4.44	1.93	NS	NS
*Propionibacterium*	6.17	4.76	NS	NS
Bacteroidetes	9.4	16.0	NS	NS
*Prevotella*	2.60	6.50	NS	NS
Unclassified_*Bacteroidales*	1.06	2.09	NS	NS
Firmicutes	23.1	25.4	NS	NS
Unclassified_*Lachnospiraceae* OTU2	0.48	0.08	0.06	NS
*Bacillus*	0.48	0.04	0.10	NS
*Staphylococcus*	3.47	1.24	NS	NS
Unclassified_*Ruminococcaceae*	1.93	2.63	NS	NS
*Lactobacillus*	6.17	5.34	NS	NS
*Solibacillus*	0.68	1.93	NS	NS
*Streptococcus*	1.93	1.01	NS	NS
OD1	0.7	0.0	0.08	NS
Unclassified_ZB2	0.68	0.04	0.08	NS
Proteobacteria	51.6	46.6	NS	NS
Unclassified_*Pseudomonadaceae*	2.80	0.97	NS	NS
*Sphingomonas*	2.89	2.09	NS	NS
*Psychrobacter*	12.4	19.3	NS	NS
*Acinetobacter*	22.1	15.9	NS	NS

Only top ten genera and those with a consistent p-value (<0.1) were included in the table.

Numerical differences between groups (p < 0.1) were observed for some genera, including unclassified_*Lachnospiraceae*, which is an intestinal bacterium. Although concentration was low, this bacterium was more abundant in the CON group (0.48%) compared to the WB group (0.08%). Some distal intestinal bacteria were also detected, namely *Corynebacterium, Prevotella*, unclassified_*Bacteroidales*, unclassified_ *Ruminococcaceae* and *Lactobacillus* as well as bacteria colonizing the small intestine (*Psychrobacter, Acinetobacter*). FDR correction did not reveal any differences between treatments.

### Piglets

As insufficient amount of DNA was extracted from the meconium, only results concerning the colonic contents of the piglets at days 26/27 of lactation are presented here. The Shannon index of the microbiota from piglets’ colon content did not show any difference in diversity between treatments (boxplot displayed in Supplementary Figure SF2, p = 0.6). However, some differences in the relative abundance of genera existed and are presented in Table 3. The most abundant genera in the colon did not differ significantly between both groups but numerical differences were observed for less abundant genera. Indeed, *Colinsella spp*., a butyrate producing genus, was significantly more abundant (p < 0.05) in the CON group while *Methanobrevibacter*, unclassified_*Clostridiaceae* (FDR < 0.05) and unclassified_*Lachnospiraceae* (p < 0.05) were more abundant in the WB group. Some genera also exhibited numerical differences between treatments (p < 0.10), i.e. *Butyricimonas, Odoribacter* and *Ruminococcus* were more abundant in the CON group, whereas *Phascolarctobacterium* and *Roseburia* were more abundant in the WB group.

**Table 3:** Relative abundance of bacterial genera sampled in the colon of piglets born from sows fed the control diet (CON) and the wheat bran-enriched diet (WB), only genera with abundance >0.01% are displayed in this table.

	CON (N = 7)	WB (N = 7)	P-value	FDR
Actinobacteria	0.71	0.57	NS	NS
*Collinsella*	0.29	0.08	**0**.**04**	NS
Bacteroidetes	32.3	28.4	NS	NS
*Butyricimonas*	0.15	0.02	0.07	NS
*Odoribacter*	0.25	0.02	0.07	NS
*Bacteroides*	6.72	2.21	NS	NS
Unclassified_*Bacteroidales*	3.27	5.61	NS	NS
*Prevotella*	12.3	11.8	NS	NS
Euryarchaeota	0.01	0.02	**0**.**05**	NS
*Methanobrevibacter*	0.01	0.02	**0**.**05**	NS
Firmicutes	56.0	63.2	NS	NS
Unclassified_*Clostridiaceae*	1.57	2.82	<**0**.**001**	**0**.**04**
*Unclassified_Lachnospiraceae OTU2*	1.91	4.14	**0**.**04**	NS
*Ruminococcus*	1.74	0.85	0.07	NS
*Phascolarctobacterium*	2.35	3.68	0.07	NS
*Roseburia*	0.11	0.57	0.09	NS
*Lactobacillus*	14.8	13.1	NS	NS
Unclassified_*Clostridiales*	6.57	6.97	NS	NS
Unclassified_*Ruminococcaceae*	11.7	14.3	NS	NS

P-values and FDR are considered as significant < 0.05 and numerically different with a value < 0.1.

In piglets’ intestinal contents, SCFA were affected by the maternal dietary treatment. The molar ratio of acetate was higher in the caecum of WB piglets (57%) compared to CON piglets (51%) and the same tendency (p = 0.06) was observed in the colon (Table 4). Butyrate molar ratio was lower in the WB group in the caecum (9.73% in WB vs 13.3% in CON). Valerate molar ratio was lower in the WB group compared to the CON group for each intestinal part. Concerning BCFA, no impact of the maternal dietary treatment was observed, as isobutyrate and isovalerate concentrations were not significantly different for each intestinal part.

**Table 4:** SCFA concentrations and molar ratios of piglets’ digesta in the terminal ileum, caecum and colon. The sum is expressed in mg.g^−1^ while other compounds are expressed as molar ratios ± SEM.

Intestinal part	Treatment	Total SCFA concentration (mg.g^−1^)	Lactate (%)	Acetate (%)	Propionate (%)	Isobutyrate (%)	Butyrate (%)	Isovalerate (%)	Valerate (%)
**Ileum**	CON ( N = 5)	8.97 ± 5.11	11.1 ± 2.98	30.2 ± 8.21	28.4 ± 11.4	1.81 ± 1.49	13.0 ± 9.36	3.00 ± 1.13	12.5 ± 5.43
	WB ( N = 7)	7.51 ± 6.74	18.4 ± 17.4	31.7 ± 16.3	24.4 ± 12.9	1.38 ± 1.32	7.50 ± 5.29	10.4 ± 11.5	6.22 ± 3.38
	p-value	NS	NS	NS	NS	NS	NS	NS	**0**.**03**
**Caecum**	CON ( N = 7)	10.3 ± 0.40	2.62 ± 0.55	51.1 ± 2.20	20.3 ± 0.73	2.77 ± 0.12	13.3 ± 1.19	3.70 ± 0.50	6.29 ± 0.73
	WB ( N = 7)	9.41 ± 0.84	3.45 ± 0.57	57.5 ± 1.27	19.4 ± 0.52	2.70 ± 0.17	9.73 ± 0.85	3.05 ± 0.19	4.22 ± 0.58
	p-value	NS	NS	**0**.**03**	NS	NS	**0**.**03**	NS	**0**.**04**
**Colon**	CON ( N = 8)	7.31 ± 1.26	9.70 ± 2.77	41.5 ± 4.15	19.6 ± 1.28	5.05 ± 2.57	13.3 ± 1.62	3.48 ± 0.33	7.42 ± 0.73
	WB ( N = 8)	7.08 ± 0.67	7.28 ± 1.14	51.2 ± 2.28	19.3 ± 0.55	2.38 ± 0.15	11.9 ± 0.92	3.32 ± 0.27	4.63 ± 0.55
	p-value	NS	NS	0.06	NS	NS	NS	NS	**0**.**01**

Results of Pearson’s correlations between SCFA ratios and microbiota data of the colon are shown in Table 5. Only results with r > 0.70 and p < 0.05 were included in this table, while other significant correlations can be found in the Supplementary Table ST5. *Butyricimonas*, which differed between treatments, was positively correlated with butyrate production (r = 0.80) which also showed a treatment effect. The same observations, but then negatively, occurred for *Phascolarctobacterium* and valerate production (r = −0.73). Other correlations were found between butyrate production and unclassified_*Rikenellaceae, Butyricimonas*, *Faecalibacterium*, *Catenibacterium*, unclassified_*Fusobacteriaceae*, *Fusobacterium, Bilophila* and *Flexispira* (r > 0.80).

**Table 5:**
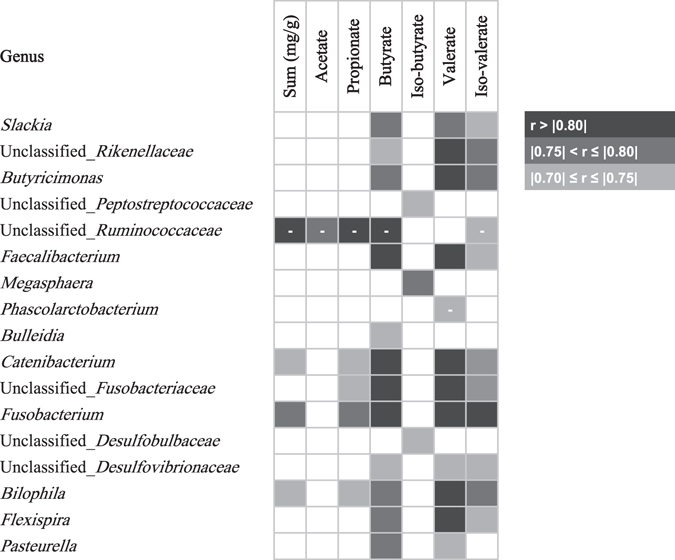
Pearson’s correlations between SCFA molar ratios and genera of the microbial community in the colon of piglets from sows fed a control and a wheat bran-enriched diet (N = 14). Only the results with a p-value < 0.05 and r > 0.70 were included in this table. Negative correlations are expressed in the table with the symbol “-”.

## Discussion

The aim of the study was to investigate whether the addition of wheat bran in the diet fed to gestating and lactating sows would alter their intestinal microbiota and if this diet would in turn alter their offspring’s microbiota and subsequent SCFA production. Moreover, the study aimed at determining whether a maternal transfer of microbiota occurred already *in utero*. Regarding the latter and to the author’s best knowledge, it is the first time in pigs that a maternal transfer occurring during the gestation period is reported, as shown by the umbilical cord blood microbial results. Similar results have been observed in humans as Jimenez *et al*.^13^ isolated four bacterial genera from the umbilical cord blood: *Enterococcus, Propionibacterium, Staphylococcus*, and *Streptococcus*. The three last genera had also a high share in the microbial communities of umbilical blood in this experiment with pigs, strengthening the reliability of our results. In this experiment, more than four genera have been observed, which is probably due to the direct DNA extraction from blood instead of the pre-culturing of blood that was done by Jimènez *et al*.^13^. As no bead beating step was added for the umbilical blood, it may be possible that more bacterial DNA could be extracted by adding bead beating steps during the DNA extraction. The presence of intestinal bacteria in the umbilical cord blood suggests a microbial transfer from the mother to the offspring already during gestation. So it can be surprising that no detectable DNA was found in the meconium. A first explanation for this lack of detectable DNA in the meconium is that some bacteria found in the umbilical cord blood are not hosted in the colon lumen (i.e. meconium collected) but in the small intestine like *Psychrobacter* and *Acinetobacter* ^25^ or in the intestinal mucus layer, like *Proteobacteria* and *Ruminococcaceaeae* ^26^ and could thus have colonized these locations that were not sampled in this study. Also, as the kits used for blood and meconium DNA extractions were not the same, it might be that the kit used for meconium may not be sensitive enough to quantify small amounts of DNA. Other evidences of maternal bacterial transfer during gestation could be found by the analysis of mucus layer and small intestine of the piglets and the use of a more sensitive kit to extract DNA from the meconium. To understand better the mechanism of the maternal transfer during the gestation period, sampling of amniotic fluid that is directly ingested by the foetus in the second part of the gestation^27^ could be interesting in the future as well as placenta during C-section.

Regarding the main hypothesis in this experiment, namely the introduction of WB into sows’ diet and the impact on their microbiota, it was shown that during gestation, 13 genera abundances differed in sows’ faeces between both dietary groups and 2 additional genera differed during lactation. Only during gestation (G98+), a nice clustering of the two groups was observed with the PCoA, most probably due to the genera for which p-values were different.

Ivarsson *et al*.^9^ found that lactic acid bacteria in faeces of growing pigs fed a 14% WB diet were significantly higher while *Prevotella* were lower. This tendency was not observed in the current study for *Prevotella* while *Lactobacillus* relative abundances were higher during G98+ and L in the WB group without being statistically significant, probably due to a high variability between individual sows, which is in agreement with studies highlighting the individual variations^28^. Seen the variability between individuals, it can be interesting for future research to increase the number of animals with the same genetic background and equal parity. In this study, results showed a higher proportion of *Oscillospira*, which has been reported to be a butyrate-producing genus^29^, in the CON group during gestation. However, a negative correlation (see Supplementary Table ST5, r = −0.57) has been observed between butyrate production and the abundance of *Oscillospira* for piglets, suggesting that this genus can have a different impact on butyrate production depending on the species or strains. Most of the differences in sows’ faecal microbiota occurred for minor groups of microbiota present in the faeces of sows (<1% of the total microbiota). However, some genera with a different abundance amongst treatments were well-represented in the microbial community, i.e. *Oscillospira* (2.7% in the CON group, 1.8% in the WB group), the unclassified *Bacteroidales* (6.1% in CON, 2.2% in WB) and *Clostridiales* (7.2% CON, 5.4% WB). During the lactation period, the butyrate-producing genus *Butyrivibrio* was increased in the WB group (0.14%) compared to the CON group (0.04%). Different studies^30–32^ showed an increase in *Bifidobacterium* in the distal part of the gastrointestinal tract of growing pigs fed by a WB diet, but this was not observed for sows in the present study. It must be emphasized that most studies on WB have been performed on piglets or growing pigs, which renders the comparison difficult as the microbiota is related to age^22^ and encounters changes as the pig grows^28^.

The differences in the microbial composition of sows’ faeces observed for the two dietary treatments in gestation were less pronounced in lactation, where only 2 genera were different between both groups, illustrated by the lack of clustering between groups for PCoA in opposition with gestation. The first possible explanation for this resides in the lower amount of WB in the diet that could be included to meet the nutritional requirements of the sows during the lactation period. A second hypothesis is that the gestation and lactation diets contained different ingredients in different proportions, such as soya pods that can be fermented. Thirdly, microbiota composition is not stable and can vary with physiological stages. It is particularly true for piglets for which colonization by microbiota has been demonstrated to be affected by stress^33^ but may apply to sows as farrowing and lactation are stressful periods due to handlings on sows and piglets and accompanied with physiological changes. A last explanation could be the different environment in gestation and lactation, as the bedding materials differed.

The maternal dietary treatment impacted the composition of the microbiota in piglet’s colon, which was distinct from the sow’s faecal microbial alterations. This was also observed by different studies when feeding sows with inulin or probiotics^11, 12^. As highlighted by Paßlack *et al*.^12^, the discrepancy between microbiota changes in sows and piglets can be ascribed to the use of faeces for sows and colon content for piglets, which may not be the exact reflection of a sow’s colon. Another explanation for these differences resides in the fact that as diet is a major driver for microbiota composition, the microbiota is probably the reflection of their different diets (solid diet for sows *vs* milk for piglets). Moreover, the piglet acquires not only the faecal microbiota from the sow but also microbial communities present in the vagina, on the skin and in the environment of its mother. All these observations probably contributed to some extend the discrepancies between sow’s and piglet’s microbiota. Furthermore, besides a faecal transfer, the colostrum and the chemical and microbial composition of milk might as well influence the intestinal microbiota of the progeny^22^ which is worth to be investigated. As microbiota composition is related to age, it would be interesting to analyse pigs’ microbiota at the adult age to see if and how these discrepancies evolve, as a stable microbiota was not reached at our sampling time period^22^ (26 days of age).

SCFA production in piglets’ intestinal contents was measured and differences between groups were observed. In the caecum and colon, acetate was higher for WB piglets compared to CON animals, whereas valerate was lower for WB piglets for each intestinal part. The butyrate production was higher in the CON pigs in the caecum, which was unexpected but no support in literature could be found on indirect impact of maternal WB on progeny’s butyrate production. Some plausible explanations for these results can be found in the correlation matrix between microbial genera and SCFA production in the colon. Indeed, the lower valerate production in the WB group can be partly explained by the higher abundance of genera with a negative correlation with valerate production: the unclassified_*Bacteroidales* (r = −0.55), the unclassified_*Clostridiaceae* (r = −0.63), unclassified_*Lachnospiraceae* OTU2 (r = −0.58) and *Phascolarctobacterium* (r = −0.73). Moreover, some valerate-producing genera, as determined by the correlation matrix, were more abundant in the CON piglets than in the WB: *Colinsella* (r = 0.57) and *Odoribacter* (r = 0.69). *Butyricimonas* was positively correlated with butyrate (r = 0.80), iso-valerate (r = 0.75) and valerate (r = 0.86) production, explaining partly the higher valerate and butyrate production of the CON piglets as this genus was significantly more abundant than in the WB piglets. The lower valerate concentration could be considered as beneficial for health, as valerate is an end-product of protein fermentation and can lead to the production of toxic compounds, even though the impact of valerate on the colon is poorly documented^34–37^. The higher acetate production in the WB group does not seem to increase butyrate production (bacteria can use acetate as alternative pathway to produce butyrate) but can be further absorbed by colonocytes as energy source and taken up by the liver for energy purposes^7^.

## Conclusion

In conclusion, this study showed that a maternal transfer is possible, and that it might already take place during gestation, as seen by the microbiota composition of the umbilical cord blood. The maternal diet impacted the piglet’s microbiota and fermentation end-products profile, even though, conversely to what was expected, the butyrate did not increase in the WB piglets. In a more holistic approach for future studies, it would be interesting to investigate long-term effects on piglet’s microbiota and health.

### Data availability

Raw sequences can be found on the ENA (European Nucleotide Archive) database, under the project accession number ERP023150.

## Electronic supplementary material

Supplementary tables and figures
