# Supplementary material for: Modulation of piglets’ microbiota: differential effects by a high wheat bran maternal diet during gestation and lactation
Source: Sci Rep. 2017 Aug 7;7:7426. doi: 10.1038/s41598-017-07228-2 (PMC5547061; doi:10.1038/s41598-017-07228-2)
Supplement: Supplementary file 1 — Supplementary tables and figures [file 41598_2017_7228_MOESM1_ESM.pdf]

Modulation of piglets' microbiota: differential effects by a high wheat bran maternal diet during gestation and lactation

Supplementary material

Julie Leblois[1,2], Sébastien Massart[3], Bing Li[1], José Wavreille[4], Jérôme Bindelle[1], Nadia Everaert[1*]

[1]Gembloux Agro-Bio Tech, TERRA, Teaching and Research Centre, University of Liège, Precision Livestock and Nutrition Unit, 5030 Gembloux, Belgium
[2]Research Foundation for Industry and Agriculture, National Scientific Research Foundation (FRIA-FNRS), Brussels, Belgium
[3]Gembloux Agro-Bio Tech, Laboratory of Urban and Integrated Plant Pathology, TERRA, Teaching and Research Centre, 5030 Gembloux, Belgium
[4]Walloon Agricultural Research Centre, Production and Sectors Department, 5030 Gembloux, Belgium
*nadia.everaert@ulg.ac.be

Supplementary Table ST1. Composition and nutritive values of sows' diets.

Ingredients (%)	GC	GWB	LC	LWB
Wheat	19.993	15.3	23.954	17.942
Maize	15	12	12	12
Barley	15	10	11.4	10
Wheat bran	-	25	-	14
Soya	-	-	13.7	13.1
Bread flour	6.5	5	6.5	5
Sugar beet pulp	5.5	5.5	5.5	5.5
Biscuit flour	5	3.5	5	4
Cocoa pods	5	1.5	3.9	0.8
Sunflower meal	9	4.2	2.5	1
Palmist meal	4	4	1.2	1.6
Soya pods	1.9	1.9	3	3
Treacle	2	1	3	3
Nutex 68 (Dumoulin Inc)	3.6	3.3	3.3	3.3
Chalk	1.17	1.21	1.61	1.66
Lard	0.47	1.25	1.47	2.25
Rapeseed meal	3	2.2	-	-
Rapeseed flour	1.2	1.2	-	-
Soya oil	-	0.55	0.05	-
Minerals & Vitamins	1.242	0.982	1.41	1.34
L-Lysine 50 %	0.386	0.372	0.393	0.385
L-threonine	0.039	0.038	0.081	0.09
DL-methionine	-	-	0.032	0.033
Thr+Met 70/30	-	0.034	-	-
DM	88.78	88.82	88.3	88.3
Crude ash	5.91	5.71	6.24	6.07
OM	94.09	94.29	93.76	93.93
CP	14.59	14.55	18.09	17.4
ADF (%)	11.89	11.43	9.88	9.75
NDF (%)	22.41	25.41	19.73	20.93
Starch (%)	34.06	30.56	34.21	31.15
Fat (%)	5.53	7.22	6.12	6.97
GE (kcal/kg MS)	4520	4633	4508	4613

WB = wheat bran; GC = gestation, control diet; GWB = gestation, WB diet; LC = lactation, control diet; LWB = lactation, WB diet
DM = analytical dry matter; OM= organic matter; CP = crude protein; GE = gross energy; ADF = acid detergent fiber; NDF = neutral detergent fiber

Supplementary Table ST2. Piglets' creep feed composition.

Ingredient (%)	Creep feed
Maize flaked	44.9
Skimmed milk	23.5
Soybean	10.0
Soybean meal	10.0
Maize starch	5.0
Soybean oil	2.2
Vit:min	1.0
Monocalciumphosphate	0.9
Cellulose	0.8
Inert markers	0.5
L-lysine HCl	0.4
Salt	0.3
Phytase	0.2
L-Threonine	0.1

Supplementary Table 3. Composition of the faecal microbiota of sows before the diet change (G21), expressed as a percentage (%) of the total microbiota. Only genera with a relative abundance >0.01% were included in this table.

	CON	WB	P-value	FDR
**Bacteroidetes**				
Prevotella	15.3	20.3	NS	NS
Unclassified_*Lachnospiraceae*	6.92	6.97	NS	NS
**Firmicutes**				
Unclassified_*Ruminococcaceae*	16.4	19.9	NS	NS
Lactobacillus	15.3	7.8	**0.002**	NS
Unclassified_*Clostridiales*	6.91	6.81	NS	NS
Ruminococcus	2.68	2.55	NS	NS
Blautia	0.17	0.32	0.04	NS
Bacteroides	0.11	0.02	0.03	NS
Bulleidia	0.07	0.13	0.03	NS
**Proteobacteria**				
Treponema	2.92	2.12	0.08	NS
Phascolarctobacterium	1.93	1.88	NS	NS
Paraprevotella	0.02	0.00	0.01	NS
**Spirochaetes**				
Unclassified_*Bacteroidales*	4.75	3.66	NS	NS
Oscillospira	2.76	2.30	NS	NS
**TM7**				
Unclassified_*Enterobacteriaceae*	0.09	0.01	0.07	NS
**WPS-2**				
Sphaerochaeta	0.32	0.15	0.05	NS

Supplementary Table ST4. Sows' faecal SCFA production. The individual SCFA are expressed as molar ratios. Results are mean ± SEM.

Period	Treatment	N	Sum (mg.g^{-1})	%lactate	%acetate	%propionate	%isobutyrate	%butyrate	%isovalerate	%valerate
G21	CON	7	6.96 ± 0.94	2.56 ± 1.08	58.6 ± 3.54	20.83 ± 1.64	2.41 ± 0.38	10.4 ± 1.27	2.98 ± 1.10	2.24 ± 0.64
	WB	8	7.71 ± 1.75	2.48 ± 1.99	57.9 ± 2.35	20.8 ± 2.45	2.10 ± 0.73	11.9 ± 1.68	2.57 ± 1.27	2.29 ± 0.77
G98+	CON	7	8.71 ± 1.66	2.43 ± 1.45	57.0 ± 2.97	22.3 ± 3.62	1.95 ± 0.61	11.1 ± 1.97	2.58 ± 1.18	2.73 ± 0.76
	WB	8	8.50 ± 1.28	2.86 ± 1.18	56.5 ± 4.83	21.1 ± 1.95	2.13 ± 0.85	11.1 ± 0.71	3.24 ± 1.60	3.11 ± 0.83
L	CON	7	11.3 ± 2.42	1.88 ± 0.95	57.4 ± 1.72	21.4 ± 1.03	2.38 ± 0.51	10.9 ± 1.21	3.50 ± 0.90	2.53 ± 0.58
	WB	8	12.1 ± 1.97	1.84 ± 0.97	55.9 ± 1.37	23.5 ± 1.62	2.24 ± 0.22	10.9 ± 1.34	3.14 ± 0.72	2.45 ± 0.37
		ddl								
P-values	T	1	0.413	0.721	0.303	0.715	0.604	0.240	0.916	0.450
	P	2	<.0001	0.199	0.241	0.014	0.498	0.732	0.194	0.182
	T*P	2	0.389	0.835	0.864	0.050	0.547	0.078	0.446	0.340

G21: gestation before diet change, G98+: lactation after diet change, T= treatment, P= period

Supplementary Table ST5.Pearson's correlations between SCFA molar ratios and genera of the microbial community in the colon of piglets from sows fed a control and a wheat bran-enriched diet (n=14). Only the results with a p-value<0.05 were included in this table. Negative correlations are expressed in the table with the symbol "-".

Genus	Sum (mg/g)	Acetic Acid	Propionic acid	Butyric acid	Isobutyric acid	Valeric acid	Isovaleric acid
Unclassified_Microbacteriaceae		-					
Leucobacter		-					
Propionibacterium							-
Collinsella							
Slackia							
Unclassified_Rikenellaceae							
Unclassified_[Barnesiellaceae]							
Butyricimonas							
Odoribacter							
Unclassified_[Paraprevotellaceae]		-					
Unclassified_Stramenopiles		-					
Leuconostoc	-		-	-		-	-
Unclassified_Clostridia		-					
Unclassified_Clostridiales	-	-	-				
Unclassified_Christensenellaceae	-		-	-		-	
Christensenella							
Unclassified_Clostridiaceae						-	
SMB53	-	-	-	-			
Unclassified_Lachnospiraceae							
Coprococcus					-		
[Ruminococcus]					-	-	
[Clostridium]							
Unclassified_Ruminococcaceae	-	-	-	-		-	-
Anaerotruncus							
Clostridium							
Faecalibacterium							
Oscillospira				-			
Megasphaera							
Phascolarctobacterium						-	
Veillonella							
Anaerococcus							
Bulleidia							
Catenibacterium							
L7A E11	-	-					
[Eubacterium]							
Unclassified_Fusobacteriaceae							
Fusobacterium							
Sphingomonas							
Sutterella							
Unclassified_Desulfobulbaceae							
Unclassified_Desulfovibrionaceae							
Bilophila							
Flexispira							
Helicobacter							
Klebsiella							
Pasteurella							
Sphaerochaeta						-	-
Treponema			-				
Unclassified_RFP12	-			-		-	

\|0.51\|< r <\|0.60\|

\|0.61\|< r <\|0.70\|

\|0.71\|< r <\|0.80\|

\|0.81\|< r <\|0.90\|

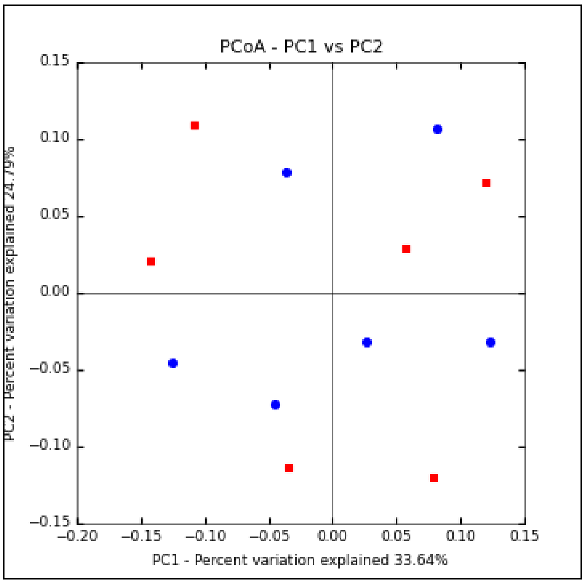

Supplementary Figure SF1. PCoA of microbial communities of sows fed the control diet (CON, N=6) and the wheat bran-enriched diet (WB, N=6) during lactation. Individual WB sows are displayed in red and CON sows in blue.

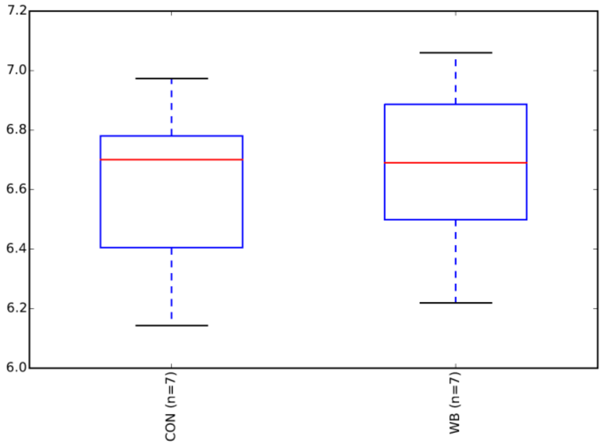

Supplementary Figure SF2. Boxplot based on Shannon index for the umbilical cord blood microbial results.

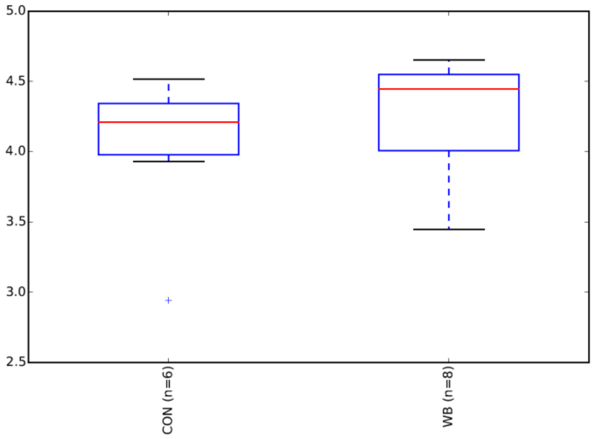

Supplementary Figure SF3. Boxplot based on Shannon index for the piglets' microbial results.
